# First Molecular Detection of *Neobenedenia girellae* (Monogenea: Capsalidae) in Farmed Greater Amberjack (*Seriola dumerili*) in Greece

**DOI:** 10.3390/ani16193139

**Published:** 2026-10-07

**Authors:** Dimitrios Karagiannis, Anna Maria M. Anastasiou, Dimitrios K. Papadopoulos, Maria V. Alvanou, Ioannis A. Giantsis, Konstantinos Feidantsis

**Affiliations:** 1 Laboratory of Aquaculture and Aquatic Animal Diseases, School of Veterinary Medicine, Aristotle University of Thessaloniki, 541 24 Thessaloniki, Greece; 2 Laboratory of Ichthyology & Fisheries, Faculty of Agriculture, Forestry and Natural Environment, Aristotle University of Thessaloniki, 541 24 Thessaloniki, Greece; aanastar@agro.auth.gr (A.M.M.A.); dkpapado@bio.auth.gr (D.K.P.); malvano@agro.auth.gr (M.V.A.); igiants@auth.gr (I.A.G.); 3 Department of Fisheries & Aquaculture, School of Agricultural Sciences, University of Patras, 265 04 Mesolonghi, Greece

**Keywords:** ectoparasite, farmed fish, monogenean infection, Mediterranean aquaculture, molecular phylogeny, biosecurity

## Abstract

In the present study, we report the first molecular detection and identification of the ectoparasitic monogenean *Neobenedenia girellae* in the farmed greater amberjack (*Seriola dumerili*) in Greece. This parasite is considered one of the most economically important ectoparasites affecting marine finfish aquaculture worldwide. The greater amberjack is an emerging species for Mediterranean aquaculture, characterized by rapid growth and high market value. During routine surveillance at a marine fish farm in the Ionian Sea, metazoan ectoparasites were collected from the sampled greater amberjack exhibiting skin and ocular lesions, and thereafter were subjected to molecular identification. Partial sequences of three genetic markers, *mitochondrial cytochrome b* (*cyt b*), *histone H3*, and *28S rDNA*, were analyzed. Phylogenetic analyses placed the Greek sequences within *N. girellae* lineages containing isolates from geographically distant regions, with no clear geographic clustering across the three molecular markers examined. Although these findings indicate genetic affinities with previously characterized isolates, they do not allow for the determination of the geographic origin or route of introduction of the parasite into Greece. Surveillance is therefore warranted to determine its distribution in Mediterranean aquaculture and decrease the risk of further spread.

## Simple Summary

Marine aquaculture is expanding rapidly, and the farming of new fish species is becoming increasingly important for the diversification of Mediterranean aquaculture. The greater amberjack (*Seriola dumerili*) is considered a promising species because of its rapid growth, high-quality flesh, and strong market value. However, diseases caused by parasites can limit its successful production. In this study, we investigated parasites collected from farmed greater amberjack showing extensive skin and eye lesions at a fish farm in the Ionian Sea, Greece. Using genetic methods, we identified the parasite as *Neobenedenia girellae*, a skin parasite known to affect several marine fish species and to cause significant health problems in farmed fish. This represents the first molecularly confirmed detection of *N. girellae* in farmed greater amberjack in Greece. Genetic comparisons showed that the Greek parasite was closely related to parasites previously reported from geographically distant regions, but the available data were not sufficient to determine how or from where it reached Greece. The finding highlights the need for increased monitoring of this parasite in Mediterranean fish farms. Further studies should investigate its distribution, effects on fish health, and effective control measures under Mediterranean farming conditions.

## 1. Introduction

Aquaculture is one of the fastest-growing food production sectors worldwide. In 2024, the global aquaculture production of aquatic animals exceeded 100 million metric tons for the first time, with a farm-gate value of approximately USD 371 billion [1]. The continuous growth of the aquaculture industry is largely driven by the need to meet the increasing global demand for high-quality animal protein and essential lipids, as the world’s population grows and capture fisheries have reached or exceeded sustainable harvest limits [2,3,4]. The Mediterranean region has a long tradition in the exploitation of marine resources, with most of this activity based on the fishing industry [5]. While fisheries production has largely stagnated due to the high exploitation of many commercial stocks [6,7,8], aquaculture continues to expand, currently producing over 2 million metric tons of aquatic animals and projected to exceed 4 million metric tons by 2030, potentially surpassing capture fisheries [9].

Marine aquaculture production is based mainly on two species: European sea bass [*Dicentrarchus labrax* (Linnaeus, 1758)] and gilthead sea bream [*Sparus aurata* (Linnaeus, 1758)]. The diversification of Mediterranean aquaculture through the introduction of new fish species is increasingly important to decrease market and economic vulnerability associated with reliance on a limited number of species [10,11]. In this context, greater amberjack [*Seriola dumerili* (Risso, 1810)] has been identified by the European Union as a promising candidate for the diversification of Mediterranean aquaculture.

The greater amberjack is a marine teleost of the family Carangidae distributed across temperate and subtropical waters of the Atlantic, Pacific, and Indian Oceans, including the Mediterranean Sea [12]. All the existing evidence suggests that it is an excellent species for farming, as it exhibits a rapid growth rate (the market size of 3–5 kg can be achieved as early as 2–3 years [13]), excellent flesh quality, and strong market demand [14,15]. Japan was the first country to produce greater amberjack commercially and became the leading producer [16]. Later and quite recently, it was developed in Mediterranean countries such as Spain, Greece, Malta, and Turkey [17].

Despite this potential, industry expansion has been constrained due to disease outbreaks induced by bacteria, viruses, and parasites. *Vibrio harveyi* (Johnson and Shunk 1936) and related vibrios are recognized as major causes of morbidity and mortality [18,19]. Furthermore, epitheliocystis caused by intracellular bacteria affecting gill and skin epithelium can be lethal at early life stages [20]. However, the parasitic infections are the most important limiting factor for sustainable production. Gill-infecting monogeneans such as *Zeuxapta seriolae* (Meserve, 1938), along with digenean blood flukes of the genus *Paradeontacylix*, have caused substantial losses in Mediterranean cage farms [21,22,23]. Skin-infecting monogeneans of the Capsalidae family represent a particularly persistent and economically important problem. Skin fluke infections have been recognized as a chronic issue in finfish mariculture since the early development of net-cage farming [24]. Most monogeneans are host-specific and can infect species belonging to the same family. For example, *Benedenia seriolae* (Yamaguti, 1934) infection has been reported in *Seriola quinqueradiata* (Temminck & Schlegel, 1845) and *S. dumerili* in Japan and in *Seriola lalandi* (Valenciennes, 1833) all over the world. *Neobenedenia girellae* (Hargis, 1955) is notable for its unusually broad host range. It has been reported from numerous farmed and wild teleost species across multiple families, including groupers [25], seabream [26], pompano [27], and Seriola species. In Japan, the greater amberjack is one of the most susceptible hosts to *N. girellae* among the farmed species [28].

*N. girellae* parasitizes the external surfaces of marine fish, including the skin and ocular region [11,26,27]. The parasite has a direct life cycle, and its transmission among susceptible fish can facilitate its persistence within aquaculture systems [11,13,28]. Infections may result in excessive mucus production, epithelial damage, erosions, ulceration, discoloration, and ocular lesions, with clinical effects potentially becoming more pronounced at higher parasite burdens [11,26,27]. Such infections may therefore have implications for fish health and production, particularly in intensive farming conditions [11,13]. The occurrence of *N. girellae* in cultured fish is also of relevance to aquaculture biosecurity, given its capacity to infect different marine fish hosts and its documented occurrence in geographically separated farming areas [11,26,27]. However, the detection of the parasite alone does not establish its source or route of introduction. Molecular confirmation is particularly valuable for reliable species-level identification, as closely related capsalid monogeneans may present similar morphological characteristics, and molecular approaches provide an additional basis for species discrimination and comparison among geographically distant populations [26,27]. The use of multiple independent molecular markers therefore strengthens species identification and provides information that can contribute to the assessment of the distribution and population structure of *N. girellae*.

Given the expanding global interest in *S. dumerili* as a diversification species for marine aquaculture, and the demonstrated capacity of *N. girellae* to compromise fish health, growth performance, and survival, a detailed understanding of the pathogenicity and epidemiology of this parasite in farmed amberjack populations is essential. Although numerous studies have investigated experimental infections of greater amberjack with *N. girellae* [24,28,29,30,31,32,33,34,35,36,37,38,39,40,41,42], information on its occurrence in commercial aquaculture facilities in Greece has not been reported to date. Thus, the present study reports the first identification (using molecular detection) of the parasite in an aquaculture facility in the Ionian Sea in Greece.

## 2. Materials and Methods

### 2.1. Sample Collection

**Figure 1:**
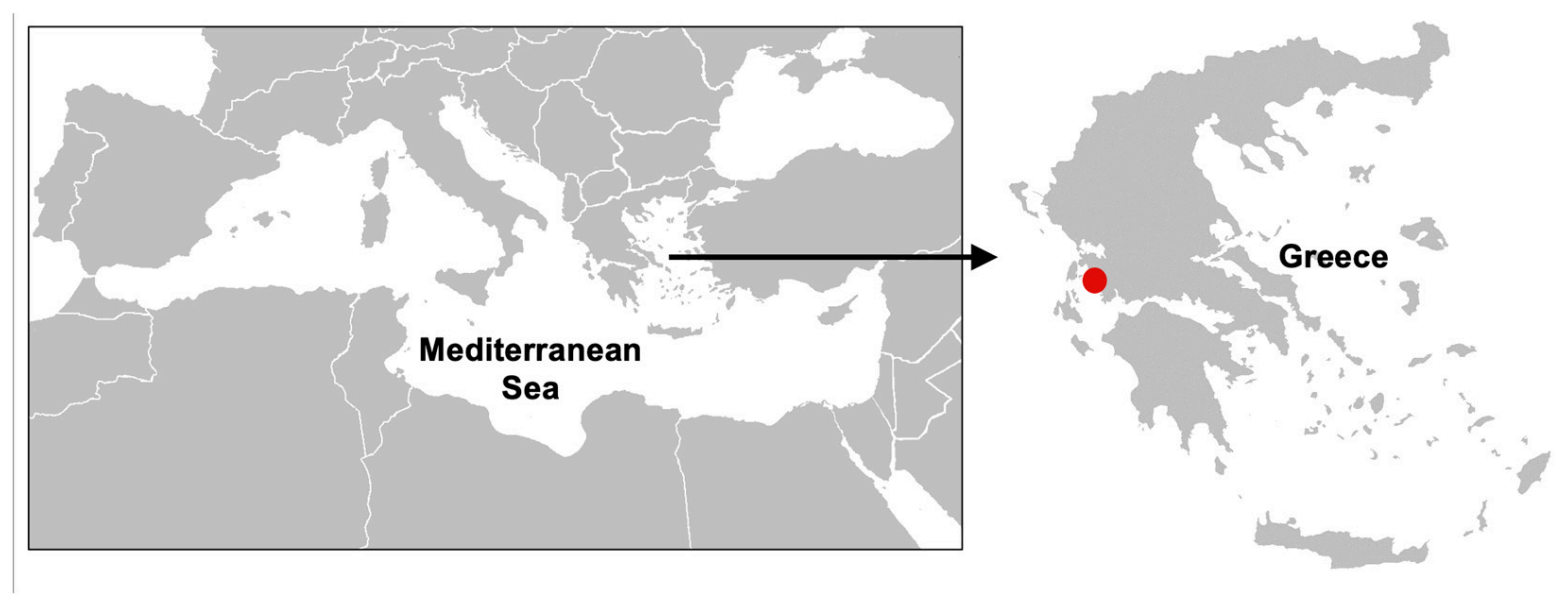
Study area in the Ionian Sea, Greece, wherefrom specimens of *Neobenedenia girellae* were collected from farmed *Seriola dumerili*.

Samplings were carried out during routine surveillance at a fish farm in the Ionian Sea (Figure 1), where 15 greater amberjack (*S. dumerili*) individuals of 249.63 g mean weight and 21.82 cm mean length exhibiting lesions were collected. The water temperature at the time of the sampling was 18.5 °C. For parasite collection, the gross lesions observed in the fish were analyzed, and the parasites that were detected were collected. Specifically, affected fish were individually examined externally under adequate illumination, with particular attention to the skin surface, head and opercular region, fins, and eyes, which are commonly parasitized by *Neobenedenia* spp. The body surface and fins were carefully inspected for attached monogeneans, while the ocular surface was examined separately for parasites associated with the eyes. The gill chambers and gills were also examined for the presence of ectoparasites. Parasites detected on the external surfaces were carefully removed using fine forceps and/or a scalpel, preserved in pure ethanol and stored at 4 °C until DNA extraction. In total, eight metazoan parasites were collected from 8 of the 15 examined fish and processed for further molecular analysis. Since sampling was targeted toward affected fish, the study cannot provide estimates of parasite prevalence or abundance, farm-level risk, or geographic distribution.

### 2.2. Molecular and Phylogenetic Analysis

Following thorough ethanol removal, whole parasites were subjected to DNA extraction using the NucleoSpin Tissue Kit (Macherey-Nagel, Düren, Germany) according to the manufacturer’s instructions. The quantity and quality of the extracted DNA were measured using a spectrophotometer (Quawell Q5000, Beijing, China), and the samples were subsequently stored at −20°C before proceeding to the next steps.

Molecular identification was based on the analysis of partial sequences of the large subunit ribosomal RNA (*28S rDNA*), histone 3 (*H3*), and mitochondrial cytochrome b (*cyt b*) genes. Three primer sets targeting these genes were used, as shown in Table 1. These genetic markers have been previously established as suitable for phylogenetic analyses of *Neobenedenia* spp. [43]. All three segments were amplified in PCRs of 20 μL total volume, composed of 10 μL FastGene Taq 2X Ready Mix (NIPPON Genetics EUROPE, Düren, Germany), 6 pmol of each primer (primer sequences in Brazenor et al.) [43], and the remaining volume ultrapure water. PCR regime was as follows: 95 °C for 3 min and then 36 cycles of 94 °C for 20 s, 58 °C for 30 s and 72 °C for 30 s, finally followed by an extension step of 72 °C for 5 min. Verification of amplification was conducted by electrophoresis in 1.5% agarose gel stained with Midori Green and visualized under a UV light system. Successfully amplified products were purified using the commercial NucleoSpin Gel and PCR Clean up kit (Macherey-Nagel, Düren, Germany), and the purified amplicons were sequenced bidirectionally applying the Sanger methodology in an ABI Prism 3730XL automatic capillary sequencer (Applied Biosystems, Foster City, California, United States) using both the forward and the reverse PCR primer of each reaction for the corresponding amplicon.

Sequences were corrected and aligned in the software package MEGA software (Version 12.1.2) [46]. Following sequence editing and alignment, the haplotypes identified for each gene were subjected in phylogenetic analyses in comparison with corresponding available haplotypes in GenBank. To determine the phylogenetic affinities of the obtained haplotypes, comparative sequences were retrieved from the National Center for Biotechnology Information (NCBI) GenBank database using the BLASTn 2.17.0 search algorithm. Sequences showing the highest similarity to the newly obtained sequences, representing a broad geographic distribution, were selected for inclusion in the phylogenetic analysis. The selected sequences were aligned using the MUSCLE algorithm, followed by phylogenetic reconstruction under the Maximum Likelihood (ML) criterion, as implemented in MEGA 12.1.2. The MUSCLE algorithm was applied as it is highly accurate for molecular identification purposes providing faster execution for standard datasets. Since the assumed probability and tree topography was expected, the Maximum Likelihood methodology was utilized because it estimates the strictest model parameters, appropriate for molecular identification. The congeneric *N. melleni* was included in the phylogenetic dendrograms in all three genes as an outgroup for better clustering depiction. The reliability of the inferred phylogenetic relationships was evaluated using 1000 bootstrap replicates. The resulting Maximum Likelihood tree was used to assess the phylogenetic relationships and genetic affinities among the newly obtained haplotypes and the comparative sequences retrieved from GenBank.

The gene sequences were submitted to Genbank and were given the accession numbers depicted in Table 2.

## 3. Results

### 3.1. Gross Examination and Parasite Detection

**Figure 2:**
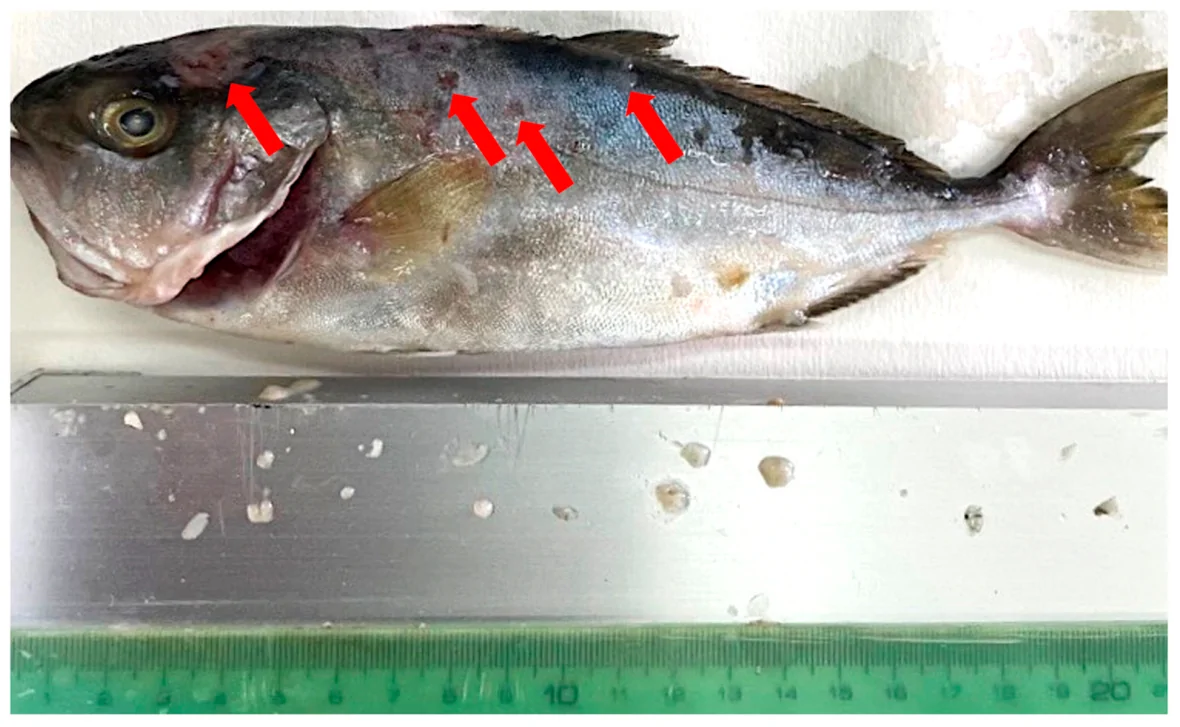
Gross lesions observed in a farmed greater amberjack (*Seriola dumerili*) from the Ionian Sea. Multifocal erosive and ulcerative skin lesions with focal hemorrhages and discoloration are evident (red arrows), particularly on the head, opercular region, and dorsal body surface. Corneal opacity is also visible.

Gross examination of the affected greater amberjack revealed multifocal erosive and ulcerative skin lesions, predominantly involving the head, opercular region and dorsal body surface. The lesions were accompanied by focal hemorrhages and areas of skin discoloration and increased mucus production. Pronounced corneal opacity was also observed in affected fish (Figure 2). A total of eight metazoan ectoparasites were collected from the examined fish and subsequently processed for molecular identification.

### 3.2. Molecular Identification and Phylogenetic Analyses

**Figure 3:**
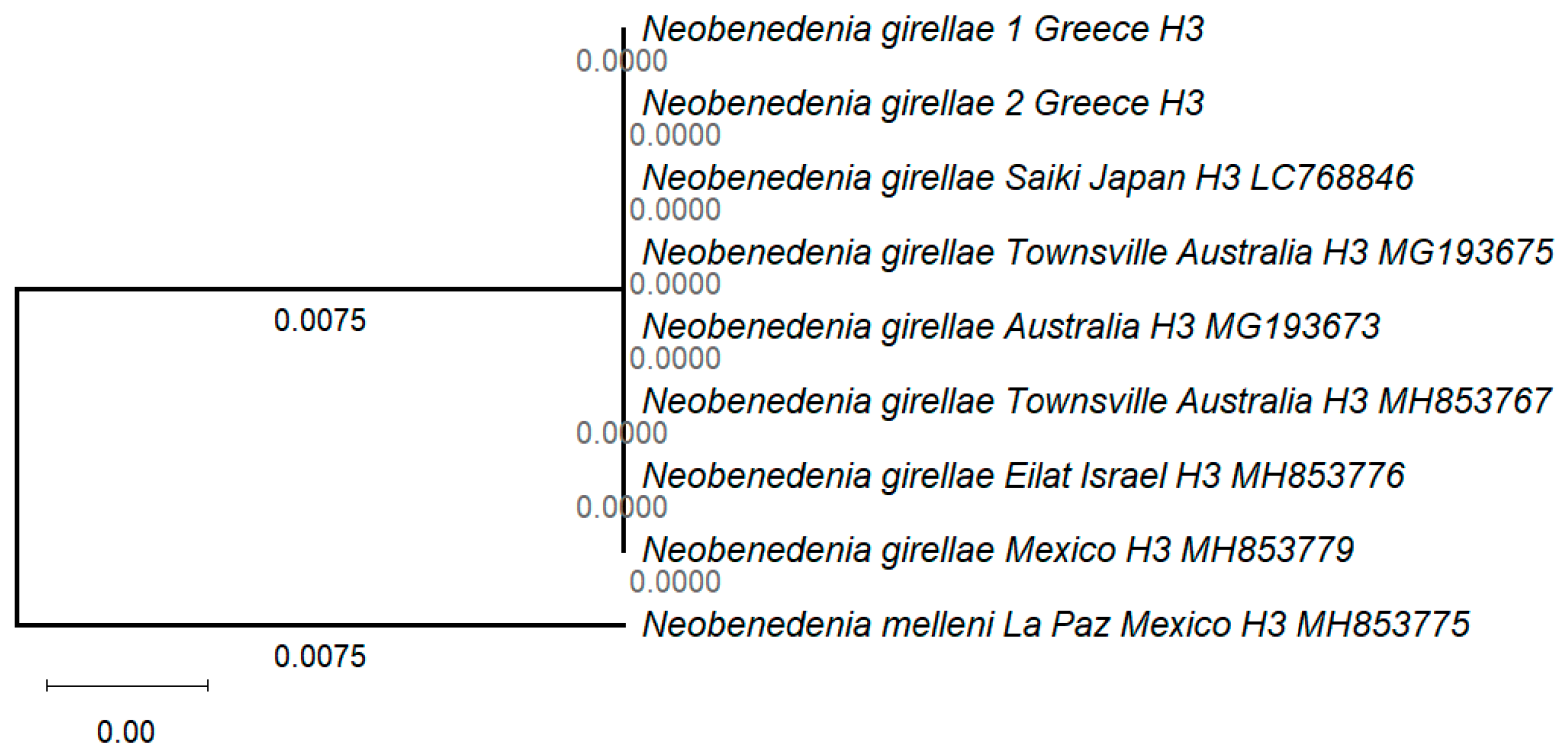
Maximum-likelihood dendrogram depicting the phylogenetic relationships among *Neobenedenia girellae* isolates collected from farmed *Seriola dumerili*, inferred from *H3* sequence data. Geographically distant *N. girellae* haplotypes included had a complete sequence similarity demonstrating the absence of genetic structure. Bootstrap values based on 1000 iterations are indicated adjacent to the nodes. *N. melleni* was used as an outgroup to better depict the genetic distances.

**Figure 4:**
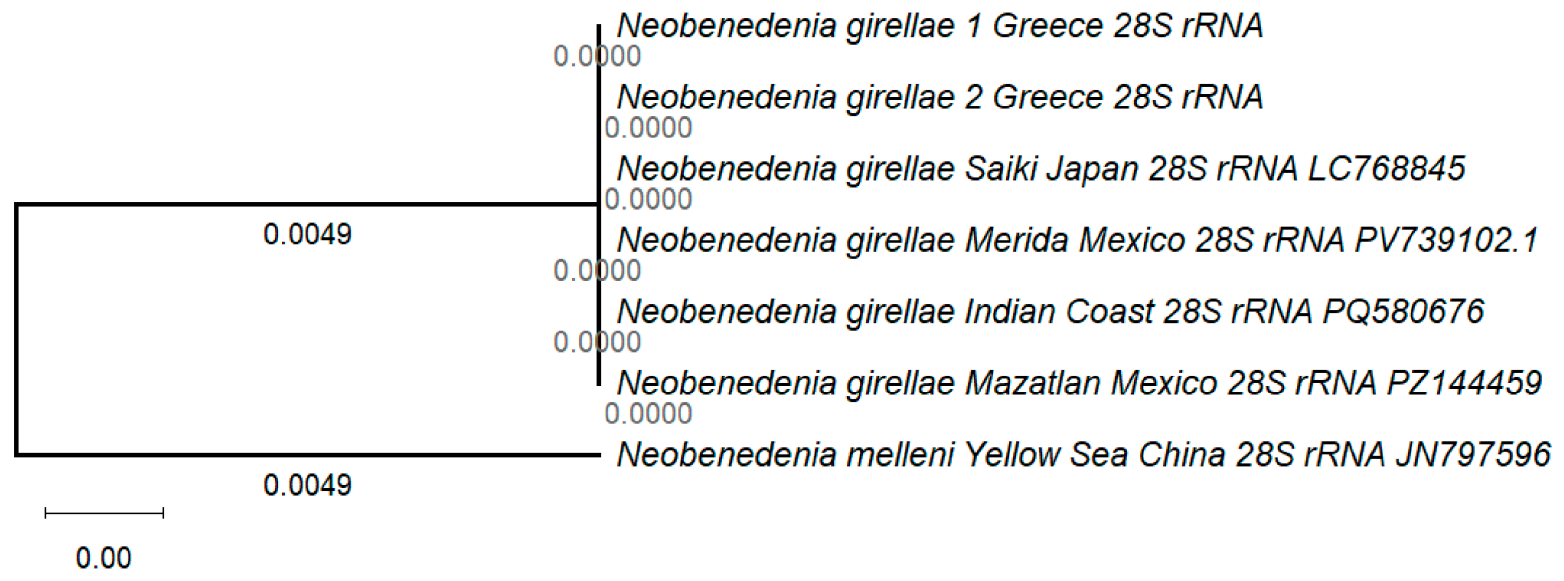
Maximum-likelihood dendrogram among *Neobenedenia girellae* haplotypes based on the *28S rRNA* gene. Geographically distant *N. girellae* haplotypes included had a complete sequence similarity demonstrating the absence of conspecific genetic structure, which was only detected among the different genus that was used as an outgroup. Bootstrap value was set as 1000, and the genetic distances are shown on the clades.

**Figure 5:**
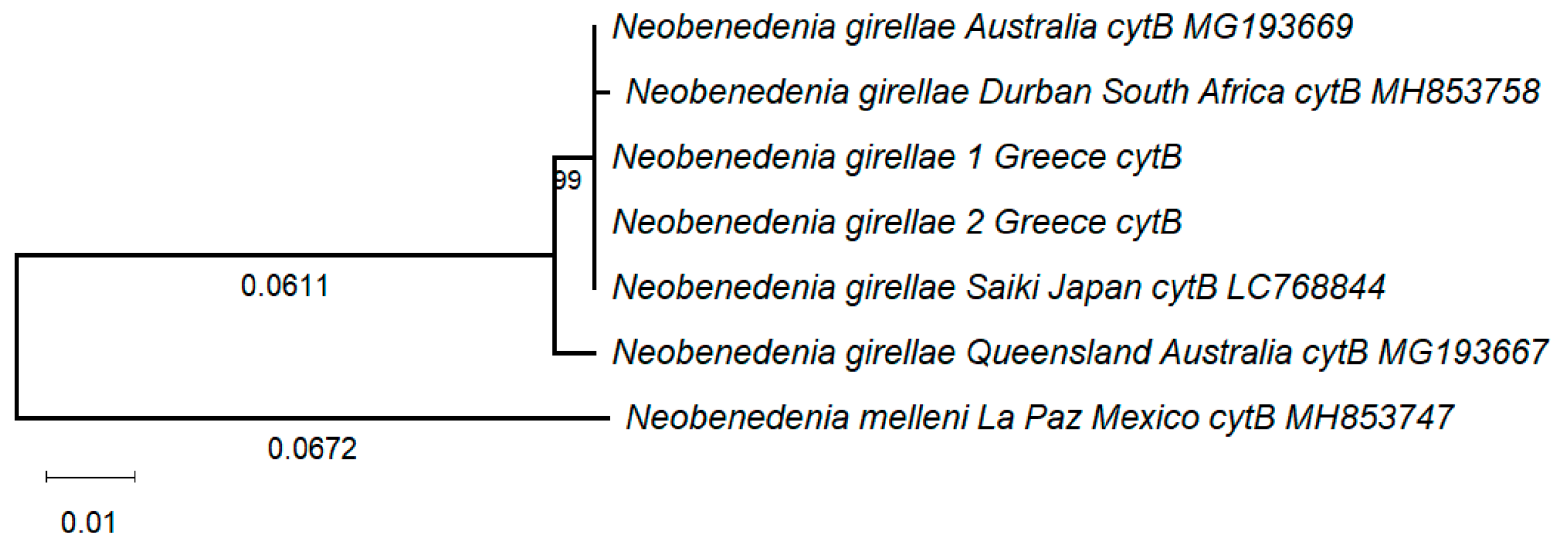
Maximum-likelihood dendrogram depicting the relationships among *Neobenedenia girellae* haplotypes based on the *cyt b* mitochondrial gene. With the exception of one haplotype from Australia, all the remining ones were characterized by the absence of genetic distance. The two conspecific groups were supported by 0.99 bootstrap value after 1000 iterations. The two Greek haplotypes were identical, as well as with the remaining ones from the same cluster.

In total, eight parasite specimens were amplified and sequenced, all of which defined only one haplotype in each of the three genes analyzed. There were no gaps or missing sites, whereas these *N. girellae* haplotypes did not form a distinct geographic lineage but were instead placed within broader *N. girellae* lineages comprising isolates from geographically distant regions. In the *H3* analysis, *N. girellae* 1 from Greece was positioned within a group containing sequences from Japan and Mexico, whereas *N. girellae* 2 from Greece was placed within the broader *H3* lineage comprising *N. girellae* isolates from different geographic regions (Figure 3). Similarly, the *28S rDNA* analysis placed both Greek haplotypes within the broader *N. girellae* lineage, together with sequences from Mexico, Japan, and the Northeast Indian Coast, rather than forming a distinct Greek cluster (Figure 4). In the *cytochrome b* analysis, both Greek haplotypes were also positioned within the broader *N. girellae* lineage. *N. girellae* 1 from Greece occurred within the lineage containing the Japanese and Australian isolates, whereas *N. girellae* 2 Greek haplotype was positioned as the closest branching sequence to the Australian *N. girellae* isolate (MG193669.1), with 100% bootstrap support (Figure 5). Sequence similarity was in all cases 100% among the Greek haplotypes with the ones retrieved from the GenBank database in all three genes analyzed. Overall, the consistent placement of the Greek haplotypes within lineages containing geographically distant *N. girellae* isolates indicates an absence of clear geographic structuring in the phylogenetic relationships observed across the three molecular markers.

## 4. Discussion

The present study confirms the occurrence of *N. girellae* in farmed greater amberjack (*S. dumerili*) in Greece. The combined analysis of three independent molecular markers (*28S rDNA*, *H3* and *cytochrome b*) with a method validated by Brazenor et al. [43] provided strong evidence for the accurate identification of the parasite. The clustering of the obtained sequences with reference *N. girellae* sequences further strengthens confidence in the species assignment. It suggests that the Mediterranean isolate does not show clear genetic differentiation from previously characterized isolates. However, a broader sampling effort would be required to properly assess genetic diversity and possible introduction pathways. To the best of our knowledge, this is the first molecularly confirmed report of *N. girellae* from farmed greater amberjack in Greece and one of the first reports from Mediterranean aquaculture facilities.

The gross lesions observed in the present case of *S. dumerili* in which *N. girellae* was detected are largely consistent with those previously described for *N. girellae* infection. Extensive erosive and ulcerative skin lesions were observed in the head and operculum region, accompanied by hemorrhagic foci, skin discoloration, and increased mucus production. Additionally, pronounced corneal opacity was evident, a finding that has also been described in *N. girellae* infection. The head has been previously identified as a particularly susceptible site of infection, possibly because cranial skin lacks scales and presents a lower density of goblet cells than dorsal skin [38]. Macroscopic erosions and ulcerations of the skin are also compatible with previous descriptions of *N. girellae* infection, in which the parasite has been associated with disruption and detachment of epidermal epithelial cells, mucus hyperproduction and leukocyte recruitment at parasite attachment sites [35,38,47]. Overall, the severity and distribution of the gross lesions observed in the present study were consistent with lesion patterns previously reported in association with *N. girellae* infection. However, because the present study was observational, a direct causal relationship between *N. girellae* and the observed lesions cannot be established.

The detection of *N. girellae* in Mediterranean aquaculture is important because it is considered one of the most significant ectoparasites affecting the culture of *Seriola* species worldwide. *N. girellae* lives on the skin surface of the fish and feeds on epithelial cells [47]. Skin damage can lead to high mortalities due to secondary bacterial infections [38,40]. Even in the absence of high mortality, infection can negatively affect feed utilization and growth [34]. Fish health and welfare are also jeopardized as a result of kidney and liver dysfunction, caused by disruption of the osmotic balance [35]. Consequently, *N. girellae* could represent an additional important limiting factor for amberjack culture, alongside (*Z. seriolae* and *Paradeontacylix* spp., which have already caused substantial losses in the region [21,22,23,48]. The pathway by which *N. girellae* was introduced into the affected farm remains unknown. Potential routes include transmission from infected wild fish occurring around sea cages [26] or the movement of infected cultured fish between production systems. Given its broad host range, *N. girellae* may also have opportunities for transmission between co-cultured or neighboring stocks of different fish species. Further epidemiological investigations are required to determine the geographic distribution of the parasite in the Mediterranean Sea.

Interestingly, gilthead seabream (*S. aurata*) is also cultured at the affected farm, but no clinical issues suggestive of *N. girellae* infection have been observed in this species to date. This contrasts with the report of Tedesco et al. [26], who identified *N. girellae* in farmed gilthead seabream in Portugal. However, as gilthead seabream at the present farm were not systematically screened for the parasite, the absence of the apparent clinical disease cannot be considered evidence of absence of infection. This observation highlights the value of targeted surveillance of other susceptible species maintained at the same farming site.

The detection of this parasite in Greek aquaculture raises additional questions regarding biosecurity. Skin flukes of the family Capsalidae have a direct life cycle with no intermediate host. *N. girellae* produces short-lived, temperature-sensitive eggs and oncomiracidia capable of rapid horizontal transmission within net cages. The water temperatures encountered during the Mediterranean grow-out season may provide favorable conditions for parasite development and transmission once established, particularly during warmer periods, as the dynamics are similar as in Japanese and other subtropical production systems. Nevertheless, this remains to be confirmed under Mediterranean farming conditions. Because the present study was based on targeted sampling of clinically affected fish from a single farm, no conclusions can be drawn regarding the prevalence or geographic extent of *N. girellae* in Greek or Mediterranean aquaculture. Nevertheless, its detection highlights the need for systematic surveillance across additional farms and regions.

In this study, the water temperature at the time of the sampling was 18.5 °C. According to Hutson et al. [49], the lower thermal tolerance of the parasite is 17 °C, the higher thermal tolerance is 35 °C, and the optimal condition is 27.5 °C, while the eggs of the parasite can hatch at 18 °C. In the study of Hirazawa et al. [30], the infection of greater amberjack with the parasite was compared at water temperatures of 20 °C, 25 °C, and 30 °C. At 20 °C, attachment remained effective, but growth, maturation, egg production, reinfection, and pathological development were slower than at warmer temperatures. The most favorable conditions were at 25 °C and 30 °C. Based on these previous experimental observations, marked pathological effects would be expected to be less pronounced at temperatures close to 18–20 °C. Nevertheless, in the present case, clinically important and extensive lesions associated with *N. girellae* infection were observed at 18.5 °C. This observation is noteworthy because extensive lesions consistent with those previously associated with *N. girellae* infection were present at a temperature close to the lower end of the parasite’s reported thermal range. However, as the present study was based on a single sampling event, no conclusions can be drawn regarding temperature-dependent pathogenicity or seasonal infection dynamics. Also, in the summer, the water temperature of the Ionian Sea usually ranges from 23 °C to 28 °C, but in recent years there were recordings of up to 29.5 °C [50,51]. These summer temperatures fall within the range reported to favor parasite development and reproduction, suggesting that warmer periods could provide particularly favorable conditions for transmission and population increase.

However, even if the temperatures between farms in Japan and the Mediterranean region are close, treatment efficacy, dosing, and welfare implications would need to be studied locally, since it has been shown that prolonged freshwater baths may not be beneficial for the Mediterranean greater amberjack [13]. Environmental conditions, farm infrastructure, and co-occurring pathogens may also differ between production regions. The concurrent presence of other significant amberjack pathogens in Mediterranean systems, including *Vibrio*-related bacteria and epitheliocystis agents [13] raises the possibility of interactive or sequential disease pressure. Skin damage caused by *N. girellae* attachment could predispose fish to secondary bacterial infections. Furthermore, additional field studies are needed to further advance our understanding regarding complex environmental conditions, as the majority of the existing evidence has been generated under controlled experimental laboratory conditions.

Moreover, the phylogenetic relationships observed in the present study revealed close genetic affinities between the Greek *N. girellae* haplotypes and sequences retrieved from the NCBI GenBank database, including isolates originating from geographically distant regions. Notably, across all three molecular markers examined, the Greek haplotypes did not form a distinct geographic lineage despite the differences in the evolutionary characteristics of the markers used. Histone 3 (*H3*) is a highly conserved nuclear protein-coding gene [52] and is therefore expected to exhibit relatively limited sequence variation among geographically separated populations. Consequently, the close phylogenetic association observed between the Greek and geographically distant *N. girellae* sequences based on *H3* is not necessarily unexpected. Nevertheless, the occurrence of a similar lack of geographic structuring in the *28S rDNA* and mitochondrial *cytochrome b* markers, which may provide greater resolution for detecting genetic differentiation than *H3* in this context, is noteworthy. The lack of clear geographic clustering observed among isolates from distant regions may be compatible with dispersal associated with anthropogenic movements of infected hosts or aquaculture-related materials. However, the present phylogenetic data cannot determine the geographic origin, direction, or specific pathway of introduction of *N. girellae* into Greece. Although anthropogenic movements could facilitate the translocation of host-associated parasites among aquaculture regions, this hypothesis cannot be tested with the present dataset. Further population-genetic analyses incorporating a larger number of isolates and samples from the potential source regions would be required to validate this hypothesis.

Future work should prioritize systematic surveillance across Mediterranean farming sites to determine the current geographic extent and prevalence of *N. girellae*. Furthermore, genetic comparisons involving a larger number of isolates from Greece, Japan, Mexico, Australia, and other established production regions could provide valuable insights into the potential introduction and subsequent distribution of the parasite in the region. In addition, controlled infection trials under Mediterranean husbandry conditions would help quantify the pathogenicity and growth impact of the parasite in the region. Trials of existing treatment protocols would inform practical management recommendations.

## 5. Conclusions

The present study provides the first molecularly confirmed detection of *N. girellae* in farmed greater amberjack (*S. dumerili*) in Greece. The identification was supported by the combined analysis of three independent molecular markers, while the extensive skin and ocular lesions observed in the affected fish were consistent with lesions previously reported in *N. girellae* infection. The detection of this parasite is of particular relevance to Mediterranean aquaculture, where greater amberjack is an emerging species with increasing production potential. Given the broad host range and direct life cycle of *N. girellae*, its presence may represent an additional health and biosecurity concern for Mediterranean finfish farming. As sampling focused on clinically affected fish, the present findings should not be interpreted as estimates of parasite prevalence, abundance, farm-level risk, or geographic distribution. Phylogenetic analyses revealed close genetic relationships between the Greek sequences and isolates from geographically distant regions, but no clear geographic structuring was observed. Therefore, the present data do not allow the geographic origin or route of introduction of the parasite into Greece to be established. Since the study was based on a single farm and targeted sampling of clinically affected fish, its findings cannot be used to estimate the prevalence or geographic distribution of the parasite. Systematic surveillance across Mediterranean aquaculture facilities, together with broader population-genetic analyses, is therefore warranted. Future field studies and controlled infection trials under Mediterranean environmental and husbandry conditions are also needed to better characterize the epidemiology, pathogenicity, seasonal dynamics, and effective management of *N. girellae* in greater amberjack culture.

## Figures and Tables

**Table 1:** Primers used for PCR amplification of *Neobenedenia* spp. isolates.

Gene	PrimerID	Sequence (′5-′3)	Forward/Reverse	Source
*H3*	G926	GACCGCYCGYAAAAGYAC	F	[44]
	G927	AGCRTGRATDGCRCACAA	R	[44]
*28SrDNA*	C1	ACCCGCTGAATTTAAGCAT	F	[45]
	D1	TGGTCCGTGTTTCAAGAC	R	[45]
*Cytb*	M1676	TGAGTTATTATTGATGTAGAGG	F	[43]
	M1677	AAAATATCAKTCAGGCTTWA	R	[43]

**Table 2:** Accession numbers corresponding to the genes characterized in *N. girellae*.

Species	Gene	Accession Number
*N. girellae*	*histone_3*	QB005060
*N. girellae*	*cytochrome_b*	QB005061
*N. girellae*	*28S rRNA*	PZ946324

## Data Availability

Data is contained within the article.
